# Epidemiological features, therapeutic strategies and long-term immunological outcomes in virologically suppressed HIV+ very late presenters

**DOI:** 10.1186/1758-2652-13-S4-P19

**Published:** 2010-11-08

**Authors:** L Martinelli, D Francisci, A Sgrelli, F Baldelli

**Affiliations:** 1 Clinic of Infectious Diseases University of Perugia, Ospedale S. Maria della Misericordia, Perugia, Italy

## Purpose of the study

In the Western world, approximately 30% of HIV-infected individuals are still very late presenters. The aim of this retrospective study is to describe epidemiological and clinical characteristics, as well as long-term immunological outcome in virologically suppressed HIV+ very late presenters.

## Methods

We reviewed the medical records of all consecutive HIV+ patients with CD4 cell count < 200/ mmc3 at presentation, who had attended our clinic between 1996-2006, and had achieved a persistent virological suppression for at least 1 year. Demographic, clinical, virological and immunological data at baseline and at follow up visits were collected. The changes in CD4+ cell count during follow up was also examined, stratifying the population according to baseline age, HIV risk factors, CD4+ cell counts, HIV viral load , HCV co-infection.

## Summary of results

Overall 164 very late presenters with a persistent virological response were examined. Caucasian and heterosexual males represent the largest part of this cohort of virological responder, very late presenter patients. IDUs and HCV coinfected patients were under-represented as compared to our HIV population, probably due to a lower adherence. Epidemiological and clinical characteristics of the study population: 61.2% had CD4+ cell count <50/µl. 123 patients started a protease inhibitor-based regimen (75%). Respectively 25% and 52% of initial NNRTI and PI-based regimen were modified during follow up (toxicity was the most common cause of switch). After 5 years of therapy a good immunological recovery (> 500 CD4 cell/µl) was observed in 30.7% and 46% of patients with baseline CD4+ cell count < 50/µl and 51-200/µl respectively.

CD4+ cell count increased even after 5 years, reaching a full immunological recovery (>700/µl) only in 17% of patients. Patients aged ≥ 50 years, IDUs and HCV co-infected had a slower and/or lower immune recovery; no significant differences in immunological response according to baseline viral load were observed.

## Conclusions

A fair immune recovery over 5 years of HAART was seen. The CD4+ cell count restoration was conditioned by baseline values, age, HCV coinfection, and a complete immunological recovery was achieved in a very limited subset of patients.

